# Impact of T cell characteristics on CAR-T cell therapy in hematological malignancies

**DOI:** 10.1038/s41408-024-01193-6

**Published:** 2024-12-03

**Authors:** Zhongfei Tao, Zuzana Chyra, Jana Kotulová, Piotr Celichowski, Jana Mihályová, Sandra Charvátová, Roman Hájek

**Affiliations:** 1 Department of Haematooncology, University Hospital Ostrava, Ostrava, Czech Republic; 2 Department of Haematooncology, Faculty of Medicine, University of Ostrava, Ostrava, Czech Republic

**Keywords:** Immunotherapy, T cells, Haematological cancer, Translational research

## Abstract

Chimeric antigen receptor (CAR) T-cell therapy has revolutionized the treatment paradigms for hematological malignancies. However, more than half of these patients cannot achieve sustainable tumor control, partially due to the inadequate potency of CAR-T cells in eradicating tumor cells. T cells are crucial components of the anti-tumor immune response, and multiple intrinsic T-cell features significantly influence the outcomes of CAR-T cell therapy. Herein, we review progressing research on T-cell characteristics that impact the effectiveness of CAR-T cells, including T-cell exhaustion, memory subsets, senescence, regulatory T-cells, the CD4^+^ to CD8^+^ T-cell ratio, metabolism, and the T-cell receptor repertoire. With comprehensive insight into the biological processes underlying successful CAR-T cell therapy, we will further refine the applications of these novel therapeutic modalities, and enhance their efficacy and safety for patients.

## Introduction

Chimeric antigen receptor (CAR) T-cell therapy, a treatment with T cells expressing antibody-based fusion proteins targeting tumor antigens, has brought tremendous breakthroughs in the treatment of hematological malignancies, including B-cell leukemia, lymphoma, and multiple myeloma (MM) [1–7]. To date, six CAR-T cell products have been approved by the Food and Drug Administration, including four products targeting CD19 and two targeting B-cell maturation antigen (BCMA) (Table 1). From observations in multicenter clinical trials, complete response/remission (CR) rates of relapsed and/or refractory (R/R) B-cell acute lymphoblastic leukemia (B-ALL), large B-cell lymphoma (LBCL), follicular lymphoma (FL), mantle cell lymphoma, and MM generated by these CAR-T cells have reached 71–90%, 39–66%, 79–94%, 67–82% and 33–73%, respectively [1, 2, 5, 7–14].

**Table 1:** Summary of the Food and Drug Administration-approved CAR-T cell products.

Product name	Commercial name	Chimeric antigen receptor structure	Cell resource	Transfection method	Approved indications (date)
Tisagenlecleucel (tisa-cel) [1, 4, 8]	Kymriah	Anti CD19-CD8α (hinge+tm)-41BB-CD3ζ	Enriched T cells	Lentivirus	-R/R B-ALL after ≥ 2 lines therapy with aged ≤ 25 years (Aug 2017) -R/R LBCL after ≥ 2 lines therapy (May 2018) -R/R FL after ≥ 2 lines therapy (May 2022)
Axicabtagene ciloleucel (axi-cel) [3, 10]	Yescarta	Anti CD19-CD28 (hinge+tm)-CD28-CD3ζ	PBMCs	Retrovirus	-R/R LBCL after≥2 lines of therapy (Oct 2017) -R/R FL after ≥2 lines of therapy (Apr 2021) -LBCL refractory to first-line therapy or relapsing within 12 months of first-line therapy (Apr 2022)
Brexucabtagene autoleucel (brexu-cel) [2, 5, 12]	Tecartus	Anti CD19-CD28 (hinge+tm)-CD28-CD3ζ	CD19^+^depleted and CD4^+^/CD8^+^ enriched T cells	Retrovirus	-R/R MCL (Jul 2020) -R/R adult B-ALL (Oct 2021)
Lisocabtagene maraleucel (liso-cel) [6, 9, 11]	Breyanzi	Anti CD19-IgG4 (hinge)-CD28 (tm)-41BB-CD3ζ	CD4^+^ and CD8^+^ T cells separately	Lentivirus	-R/R LBCL after ≥2 lines of therapy (Feb 2021) -LBCL refractory to first-line therapy or relapsing within 12 months of first-line therapy and are not eligible for HSCT (Jun 2022) -R/R CLL/SLL after ≥2 lines of therapy (Mar 2024) -R/R FL after ≥2 lines of therapy (May 2024)
Idecabtagene vicleucel (ide-cel) [7, 14]	Abecma	Anti BCMA-CD8α (hinge+tm)-41BB-CD3ζ	PBMCs	Lentivirus	-R/R MM after ≥4 lines of therapy (Mar 2021) -R/R MM after ≥2 lines of therapy (Apr 2024)
Ciltacabtagene autoleucel (cilta-cel) [13]	Carvykti	Dual anti BCMA-CD8α (hinge+tm)-41BB-CD3ζ	Enriched T cells	Lentivirus	-R/R MM after ≥4 lines of therapy (Feb 2022) -R/R MM after ≥1 lines of therapy (Apr 2024)

*tm* transmembrane, *R/R* relapsed and/or refractory, *B-ALL* B-cell acute lymphoblastic leukemia, *LBCL* large B cell lymphoma, *FL* follicular lymphoma, *PBMCs* peripheral blood mononuclear cells, *MCL* mantle cell lymphoma, *HSCT* hematopoietic stem cell transplantation, *CLL/SLL* chronic lymphocytic leukemia/small lymphocytic lymphoma, *BCMA* B-cell maturation antigen, *MM* multiple myeloma.

However, the clinical outcomes of these treatments are inconsistent and mixed. Within one year after CAR-T cell therapy, progressive disease can be observed in roughly 50% of B-cell leukemia and LBCL, 20–30% of FL, 40% of mantle cell lymphoma, and 20–40% of MM patients [2–11, 13, 15]. Meanwhile, in R/R CLL, the most common leukemia in adults, the reported CR rates of anti-CD19 CAR-T cell therapy range from 18 to 29%, which are lower than in other B-cell malignancies [2, 5, 7, 10–13, 15, 16]. Furthermore, the most common CAR-T cell-specific side effects, cytokine release syndrome and neurotoxicity, are observed in 42–100% and 2–64% of patients in CAR-T cell clinical trials [17]. Severe cytokine release syndrome and neurotoxicity (grade ≥3) can occur in up to 46 and 50% of treated patients, respectively [17]. In addition, rates of unsuccessful CAR-T cell manufacture are approximately 25% for non-Hodgkin’s lymphoma (NHL) patients, and 6.8% for B-ALL and CLL patients, which could be a significant obstacle to the treatment [18].

**Fig. 1:**
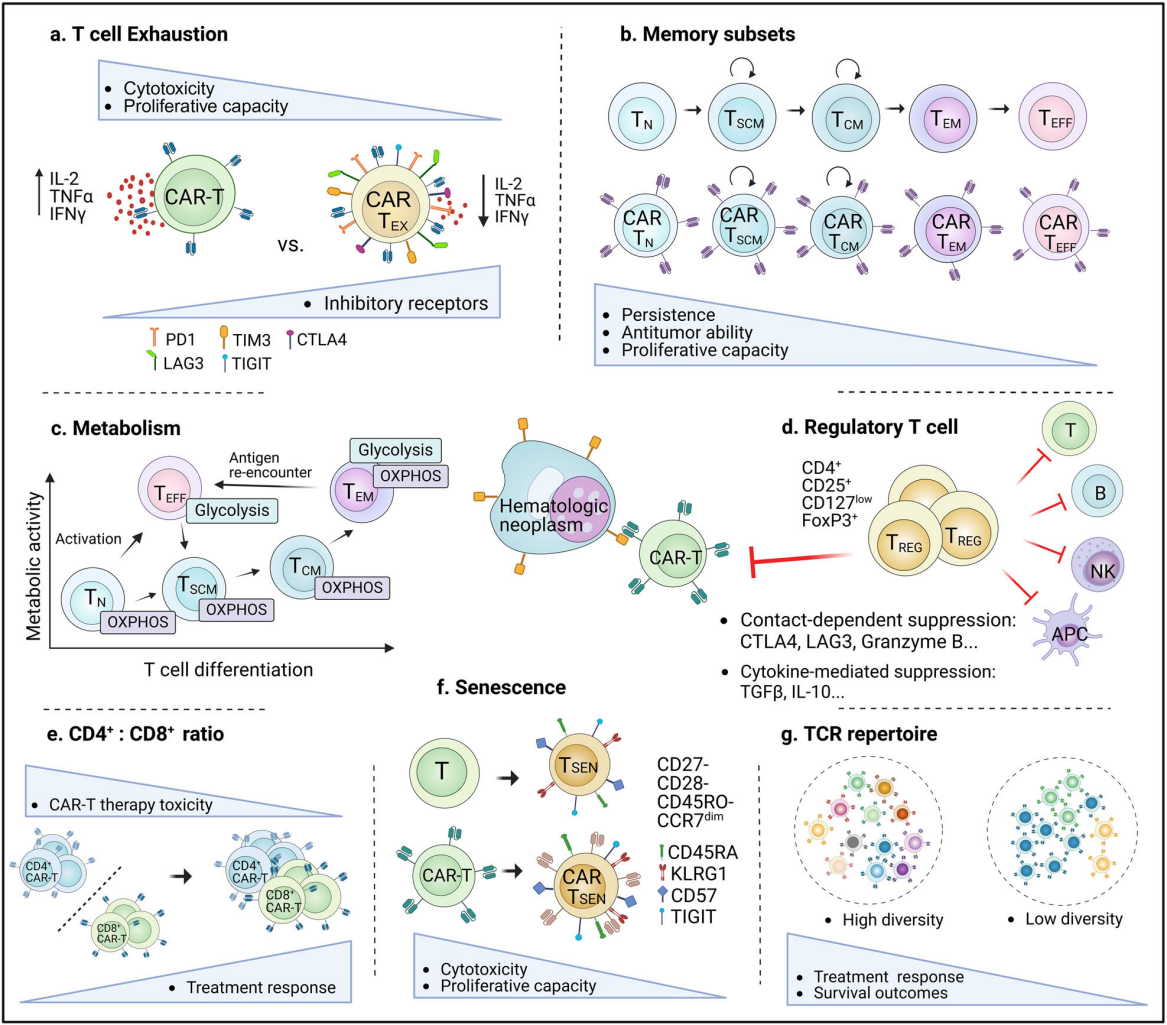
T cell characteristics associated with the efficacy of CAR-T cell therapy in hematological malignancies. **a** T_EX_ cells form during cancer where antigen persists, and are characterized by loss of cytokine production, poor proliferation, and high expression of inhibitory receptors. CAR-T_EX_ have similar features, and the exhaustion molecules are related to poor efficacy of CAR-T therapy. **b** T cells linearly differentiate to various memory subsets following cancer antigen stimulation, losing proliferation potential, but gaining effector functions. In CAR-T cell therapy, T-cell products with early memory phenotypes exhibit superior proliferative capacity, anti-tumor ability, and persistence. **c** The metabolic phenotypes and memory status of T cells are interconnected. T_N_ and early memory T cells mainly rely on OXPHOS, but shift to glycolysis as they differentiate into T_EFF._ T_EM_ utilizes both OXPHOS and glycolysis, and can rapidly increase glycolytic activity to support effector functions. **d** T_REG_ suppress the function of CAR-T cells, and their numbers are negatively correlated with treatment response and survival. **e** A defined ratio of CD4^+^/CD8^+^ CAR-T cells improves therapeutic response and reduces toxicity in CAR-T cell-treated patients. **f** Senescent CAR-T cells exhibit terminal differentiation, loss of proliferative capacity, and compromised cytotoxic function. **g** TCR repertoire can be used as a surrogate for T-cell clonotype and expansion. A highly diverse baseline TCR repertoire is associated with favorable responses and improved survival outcomes in CAR-T cell therapy. CAR, chimeric antigen receptor; T_EX_, exhausted T cells; T_N_, naïve T cells; T_SCM_, T memory stem cells; T_CM_, central memory T cells; T_EM_, effector memory T cells; T_EFF_, effector T cells; T_REG_, regulatory T cells; T_SEN_, senescent T cells; OXPHOS, oxidative phosphorylation; TCR, T cell receptor.

T cells are the most crucial immune cells in the anti-tumor immune response. T cells orchestrate the adaptive immune system by producing cytokines with chemotactic, proinflammatory, and immunoprotective properties, and also carry out direct cytotoxic reactions towards neoplastic cells [19]. CAR-T cells, which are manufactured from the patients’ T cells and capable of targeting tumor cells by circumventing major histocompatibility complex (MHC) restriction, have become the pillar of successful adoptive cell therapies. Emerging evidence indicates that multiple characteristics of patients’ T cells and CAR-T cells can significantly impact the effectiveness of CAR-T cell therapy. Herein, we summarize various aspects of T-cell biological characteristics related to CAR-T cell therapy outcomes, including exhaustion, memory differentiation, senescence, T-cell subsets, metabolism, and T-cell receptor (TCR) repertoire (Fig. 1), with the aim of improving CAR-T cell efficacy and safety profiles in hematological malignancies.

## T-cell exhaustion

T-cell exhaustion refers to post-thymic T-cell dysfunction in response to persistent antigen stimulation during chronic infections and cancer. Functionally, exhausted T cells (T_EX_) exhibit a progressive loss of cytokine production, poor proliferative capacity, and compromised killing function. Most importantly, they display high expression of inhibitory receptors, which have become the hallmarks of T_EX_. They include cytotoxic T lymphocyte-associated antigen 4 (CTLA4), programmed cell death protein 1 (PD-1), lymphocyte activation gene 3 (LAG3), T-cell immunoglobulin mucin receptor 3 (TIM3), and T-cell immunoreceptor with immunoglobulin and ITIM domains (TIGIT) [20, 21]. The gene regulatory programs governing exhaustion are largely conserved across disease settings, and epigenetic findings support T_EX_ representing a distinct T-cell chromatin state rather than merely the isolated expression of inhibitory receptors [22]. While T-cell exhaustion is indispensable to limit damage to healthy cells and prevent autoimmune diseases, it also restricts the effectiveness of cancer immunotherapy [20, 21].

The molecules related to T-cell exhaustion can serve as biomarkers to predict CAR-T cell therapy efficacy and persistence. Several studies have reported that lower frequencies of PD-1, LAG3, and TIM3 expressed by infused and engrafted CAR-T cells are correlated with better disease control [23–26]. In patients with CLL, Fraietta et al. found that those achieving CR received lower percentages of anti-CD19 CAR-T cells expressing PD-1 combined with either TIM3 or LAG3, compared with patients achieving partial or no response. They also found the presence of a group of PD-1 negative CAR-T cells (PD-1^-^CD27^+^CD8^+^) in the infusion products was associated with good response [23]. In patients with B-ALL, Finney et al. observed that engrafted anti-CD19 CAR-T cells from minimal residual disease (MRD)-negative and durable leukemia-free patients expressed lower levels of LAG3 and TIM3 compared with those from MRD-positive or non-durable leukemia-free patients [26]. In patients with LBCL, researchers confirmed that those who did not achieve an early molecular response had a higher fraction of exhausted CD8^+^ T cells in CAR-T cell infusion products, featuring increased expression of LAG3 and TIM3 [24]. Consistently, studies have also shown that higher frequencies of inhibitory receptors in leukapheresis T cells, peripheral or bone marrow T cells, or tumor microenvironment T cells correlate with worse responses in CAR-T cell-treated patients [26–28]. Furthermore, Huang et al. recently documented CD38 as another hallmark of exhaustion, co-expressed with exhaustion-related transcription factors and LAG3 in anti-CD19 CAR-T cells. Inhibiting CD38 activity could reverse the exhaustion and improve the antitumor response [29].

Interestingly, recent evidence has also suggested that CAR structures affect the exhaustion status of CAR-T cells [30, 31]. For example, incorporating a 4-1BB costimulatory domain reduced the exhaustion induced by CAR signaling, whereas a CD28 costimulatory domain augmented the exhaustion [31]. Moreover, CAR-T cells containing MYD88/CD40 costimulatory domains had lower expression of PD-1 and remained less differentiated than CD28 CAR-T cells [31]. Additionally, higher levels of tonic signaling resulting from self-aggregation of CARs have been reported to cause CAR-T cell exhaustion [30, 32, 33]. For instance, a CAR with an IgG1 CH2–CH3 region as a spacer generates stronger tonic signals than those with the CH3 region only [33]. Another type of tonic signaling activation involves self-aggregation via the framework region of the single-chain variable fragment of CAR on CAR-T cells, which impairs proliferation and promotes exhaustion [30].

Combating CAR-T cell exhaustion represents a promising therapeutic avenue with broad applicability. In addition to the abovementioned CAR structure modification, recent advances have demonstrated that PD-1 signaling interference through PD-1 antibody checkpoint blockade, CRISPR/Cas9 mediated PD-1 knockout, and anti-PD-1 antibody secreting CAR-T cells, restores the effector function of CAR-T cells [34–36]. In clinic, the feasibility and safety of anti-CD19 CAR-T cells combined with a PD-1 inhibitors (pembrolizumab or nivolumab) in patients with R/R B-ALL and NHL were confirmed in different studies [37, 38]. Some other engineering strategies have focused on restoring activator protein 1 (AP-1) function to prevent T-cell exhaustion by overexpressing the transcription factor c-Jun and the basic leucine zipper ATF-like transcription factor (BATF) [39, 40]. Additionally, Weber et al. reported ameliorating tonic CAR signaling by using dasatinib, a clinically available tyrosine kinase inhibitor, which subjected CAR-T_EX_ to transient rest to restore functionality [41].

## T-cell memory differentiation

Memory differentiation of T cells is largely linear and unidirectional. After activation, circulating naive T cells (T_N_) massively divide (up to 50,000-fold) into effector T cells (T_EFF_), which disseminate broadly to eliminate antigens. After antigens are cleared, most T_EFF_ undergo apoptosis, while a small subset survive and persist for years as memory T cells (T_MEM_), providing recall responses to previously encountered antigens [20, 42]. T_MEM_ are heterogeneous, including central memory cells (T_CM_) trafficking through lymphoid tissues, effector memory cells (T_EM_) recirculating through blood and nonlymphoid tissues and surveying the sites of infection, and memory stem cells (T_SCM_) which are rare but possess the self-renewal and multipotent capacity to reconstitute the entire spectrum of T_MEM_ and T_EFF_ subsets [43]. CAR-T cell products manufactured by ex vivo expansion are predominantly composed of antigen-experienced T cells, with the two major subsets being T_EM_ and T_CM_ [44, 45].

Infusion products with a high content of early memory CAR-T cells display superior expansion, anti-tumor response, and in vivo persistence [23, 24, 44, 46–49]. For instance, Xu et al. performed an analysis in a group of lymphoma patients and found that a subset of CAR-T_SCM_ (CD8^+^CD45RA^+^CCR7^+^) within infused product positively correlated with CAR-T cell expansion in patients [44]. More specifically, Deng et al. conducted single-cell RNA sequencing of axi-cel infusion products from 24 patients with LBCL. Their data identified threefold higher frequencies of T_CM_ (CCR7^+^CD27^+^SELL^+^) subsets in patients who achieved CR than in those with partial response or progressive disease [24]. Similar results were demonstrated by Bai et al. through cellular indexing of transcriptomes and epitopes by sequencing (CITE-seq) analysis, showing that frequencies of T_SCM_ and T_CM_ from CAR-T infusion products could distinguish relapse in patients [50]. Finally, Haradhvala et al. observed that responses to tisa-cel were associated with a striking expansion of CD8^+^ CAR-T_CM_-like cell populations from the infusion product in LBCL patients [49].

Further evidence has shown that memory signatures of the premanufacture starting T cells influence the efficacy of post-manufacturing CAR-T cells [23, 51–54]. It is well-established in a murine model that CAR-T cell manufacturing from purified T_N_ and T_SCM_ populations results in longer and enhanced anti-tumor activity, and reduces the tendency of the cells to cause cytokine release syndrome [55]. In line with the lab study, clinical studies have shown comparable results. One study of tisa-cel-treated patients with LBCL and B-ALL suggested that a higher count of T_SCM_-like cells in the apheresis, characterized by CD8^+^CD45RA^+^CD27^+^ expression, was related to the efficacy of CAR-T cell therapy [51]. In another group of ide-cel-treated MM patients, a higher percentage of naive (CCR7^+^ and CD45RA^+^) and early memory (CD28^+^CD27^+^) T cells in the apheresed product was found to be statistically correlated with long-term responses [54]. Similarly, in a cohort of 182 B-cell malignancy patients, apheresis analyses confirmed that T cells significantly enriched in naive or early memory features were associated with responding patients, whereas the T_EM_ subpopulation marked the non-responding patients [52].

Intriguingly, several studies have compared the differentiation phenotypes of CAR-T cells with CD28 versus 4-1BB costimulatory domains. They revealed that more differentiated memory phenotypes make up a greater proportion of CAR-T cells containing the CD28 domain than of those possessing the 4-1BB domain [56, 57]. Moreover, another study recently revealed that the ICOS and OX40 tandem costimulatory domain resulted in less differentiated memory CAR-T cells and superior tumor control compared with CD28 and 4-1BB CAR-T cells in preclinical models [58].

Strategies to promote naive and early memory populations in CAR-T cell manufacturing are being investigated. For instance, interleukin-7 (IL-7) and IL-21 induce T_SCM_ differentiation, and together with IL-15, they promote the maintenance and expansion of T_SCM_. By contrast, IL-2 drives terminal differentiation of CD8^+^ T cells through the high-affinity IL-2 receptor [59]. On the basis of these cytokine functions, many CAR-T cell preclinical studies and clinical trials have been conducted to search for favorable CAR-T memory subsets [44, 59–61]. Another strategy to achieve early memory phenotypes is the use of pharmacological inhibitors targeting T-cell differentiation signaling pathways during the culture of CAR-T cells, such as phosphatidylinositol 3’-kinase (PI3K) inhibitors (duvelisib) and Bruton’s tyrosine kinase inhibitors (ibrutinib) [62, 63]. Other approaches have aimed at maintaining a less differentiated CAR-T cell product. They include shortening the ex vivo culture period of CAR-T cells and manufacturing CAR-T cells from patients in the early stage of cancer [64–66]. The latter approach is appealing, as it avoids the depletion of T_N_ caused by cumulative chemotherapy cycles, promotes better CAR-T expansion in vivo, and has the potential to improve survival outcomes [66–68].

## T-cell senescence

T-cell senescence is an irreversible process of adaptive immunity degeneration, characterized by terminal differentiation and loss of proliferative capacity, yet the immune cells retain their ability to perform their effector functions. Age-related senescence involves telomere shortening, whereas premature senescence is telomere-independent. Senescent T cells have distinct phenotypes, including the loss of costimulatory molecules CD27 and CD28 and expression of CD57 and killer cell lectin-like receptor subfamily G member 1 (KLRG1) [69]. They likewise exhibit a terminally differentiated phenotype with downregulation of the chemokine receptors CCR7 and CD45RO but upregulation of CD45RA [70]. Nevertheless, there is an overlap between T-cell senescence and exhaustion; for example, the novel exhaustion marker TIGIT was also found to be upregulated in classic senescent CD8^+^ T cells [71].

CAR-T cell therapy has been most successful in pediatric and young adult B-ALL patients, with a high CR rate of 90%; this is in contrast to MM patients, whose median age is over 70 and whose reported CR rate ranges from 33 to 73% [1, 13, 14]. Although there are differences in tumor biology among these indications, dysfunctional senescent T cells are suggested to be one of the reasons for the difference in CR rates [72]. It has been reported that 75% of MM patients exhibit a subpopulation of prematurely senescent T cells. These senescent T cells were KLRG1^+^CD57^+^CD160^+^CD28^-^, had low levels of PD-1 and CTLA4 phenotypes, and had normal telomere lengths [73]. Moreover, Guha et al. made an in vitro comparison of CAR-T cells manufactured from young and geriatric healthy donors. They revealed that young CAR-T cells had significantly higher transduction efficiency, improved expansion ability, and higher cytotoxicity than geriatric CAR-T cells [74]. Although most of these data indicate that T-cell senescence adversely impacts CAR-T cell therapy, the direct evidence from clinical studies on this topic is rare, and the relationships between exhaustion and senescence require further elucidation.

## Regulatory T cell

Regulatory T cells (T_REG_) play an essential role in maintaining immune homeostasis and self-tolerance. These cells are characterized by the expression of transcription factor forkhead box P3 (FoxP3), the absence of CD127 (IL-7 receptor α), and the presence of CD4 and CD25 (IL-2 receptor α), constituting a small portion of the CD4^+^ T population. T_REG_ have a distinct ability to suppress the activation, proliferation, and effector functions of a variety of immune cells, such as CD4^+^ and CD8^+^ T cells, natural killer cells, B cells, and antigen-presenting cells [75, 76]. Multiple immunosuppressive mechanisms are employed by T_REG_, including the expression of IL-2 receptor α (CD25) for sequestration of IL-2, thereby inducing effector T-cell anergy and apoptosis; the release of immunosuppressive cytokines such as IL-10, IL-35, and TGFβ; the abundant expression of inhibitory receptor CTLA4, which inhibits antigen-presenting cell function and T cell activation; and the conversion of ATP into adenosine, an immunosuppressive metabolite that compromises T-cell activation [75, 77]. However, this regulatory ability also dampens anti-tumor immune responses and favors tumor progression [76].

Recently, increasing attention has been paid to T_REG_ in CAR-T cell therapies, particularly their elevated levels in peripheral blood and infusion products, which are linked to inferior therapeutic responses [28, 49, 78–83]. For instance, in a study involving 46 R/R B-ALL patients, Pan et al. found that the T_REG_ population (CD4^+^CD25^+^CD127^low^) in peripheral blood measured both pre- and post-CAR-T cell therapy was significantly lower in the remission group than in the non-remission group. The authors also observed that T_REG_ levels were negatively correlated with relapse-free survival, overall survival, and persistence of CAR-T cells [79]. In another study, single-cell transcriptome sequencing of CAR-T cell infusions in R/R LBCL patients revealed an elevation of the CAR-T_REG_ population among non-responders [49]. This study further proved that CAR-T_REG_ suppressed the expansion of other CAR-T cells and drove relapses in an in vivo model [49]. Similar results were indicated by an independent study of LBCL patients, which showed that increased levels of early engrafted CAR-T_REG_ in patients predicted clinical progression but less severe neurotoxicity [78]. Likewise, some other researchers have observed that increased levels of T_REG_ in peripheral blood before and after CAR-T cell infusion were associated with nonresponse and inferior overall survival [28, 80–83]. Notably, it is well established that IL-2 administration can lead to a profound expansion (nearly 4-fold) of T_REG_ in humans, which hinders the efficacy of immunotherapy [84, 85]. It is also known that CAR-T cells manufactured in the presence of IL-7 and IL-15 have superior expansion and antitumor activity to those given IL-2, partly due to a smaller increase in CAR-T_REG_ [86]. Consequently, new combinations of cytokines such as IL-7, IL-15, and/or low doses of IL-2 are becoming more appealing in CAR-T cell manufacture strategies [59, 86, 87].

## CD4^+^/CD8^+^ T cell ratio

CD4^+^ and CD8^+^ T cells represent two broad classes of T cells crucial for immune responses against infection and cancers and distinguished by divergent recognition and effector mechanisms. Upon antigenic stimulation, CD4^+^ T cells undergo proliferation and secretion of cytokines that stimulate antibody responses or lead to macrophage activation via interactions with class II MHC molecules, whereas CD8^+^ T cells kill the antigen-bearing cells through class I MHC molecules [88]. CD4^+^ T cells help CD8^+^ T cells in secondary expansion, memory response, and acquisition of effector functions during chronic antigen stimulation [89–91]. Without the assistance of CD4^+^ T cells, CD8^+^ T cells become exhausted by increasing their expression of inhibitory molecules and transcriptional programs [92].

The frequency of CD4^+^ and CD8^+^ T cell subsets in the blood can differ markedly in cancer patients because of age, the effects of chemotherapy, and thymic function [93, 94]. Nevertheless, it remains feasible to manufacture CAR-T cells with a defined CD4^+^/CD8^+^ ratio, even in heavily pretreated groups of patients [94–96]. Moreover, the CD4^+^/CD8^+^ ratio generally decreases during the CAR T cell manufacturing period due to the higher expansion rate of CD8^+^ T cells compared with that of CD4^+^ T cells [97]. A recent study identified a CD4^+^/CD8^+^ ratio less than 1:3 in peripheral blood at apheresis as a risk factor for CAR-T cell manufacturing failure [98].

The CD4^+^/CD8^+^ ratio in CAR-T cell infusion products is also believed to be an important aspect of the overall therapeutic impact of CAR-T therapy. The synergistic activity between CD4^+^ and CD8^+^ CAR-T cells was first demonstrated in murine models [99–101]. In a B-cell lymphoma murine model, Sommermeyer et al. demonstrated that the infusion of CAR-T cells with a 1:1 ratio of CD4^+^and CD8^+^ outperformed unselected CAR-T cells as well as CD8^+^ or CD4^+^ CAR-T cells alone in terms of anti-tumor effect. This synergistic efficacy is suggested to be mediated by the cytokines released by CD4^+^ T cells [99]. Consistent results were also observed in murine models of melanoma lung metastasis and mammary carcinoma [100, 101]. In the clinical setting, a defined CD4^+^/CD8^+^ ratio of CAR-T cell infusion has been shown to improve therapeutic efficiency and reduce toxicity [94–96, 102]. For instance, Turtle et al. demonstrated in a study of 29 adult B-ALL patients that low doses (2×10^5^/kg) of CAR-T cells with a 1:1 CD4^+^/CD8^+^ ratio achieved an impressive 93% CR rate and an 86% MRD-negative rate [95]. Another study involving children and young adults with B-ALL reported that a defined CD4^+^/CD8^+^ ratio retained an effective response while reducing the severity of CRS [102]. In B-cell lymphoma, Galli et al. found that patients with complete or partial responses at three and six months after CAR-T cell infusion had a lower CD4^+^/CD8^+^ ratio in the infused CAR-T products compared with non-responders. They also observed that patients with a CD4^+^/CD8^+^ ratio of less than 1.12 had a three-fold higher risk of developing neurotoxicity compared to those of a higher ratio [103].

Accordingly, liso-cel, one of the Food and Drug Administration-approved commercial products, is manufactured from leukapheresis-selected CD8^+^ and CD4^+^ T cells, followed by independent CD8^+^ and CD4^+^ T cell activation, transduction, expansion, and formulated into 1:1 ratio [104]. This product can provide high response rates in patients with R/R hematological malignancies [96, 104, 105]. However, Lee et al. to raised concerns about the suboptimal expansion and hypofunction of CD8^+^ CAR-T cells when grown in the absence of CD4^+^ T cells. They suggested manufacturing and growing both CD4^+^ and CD8^+^ T cells together and then formulating the defined ratio that will be infused [106]. A direct comparative trial will be necessary to verify the clinical superiority of this fixed CD4:CD8 approach.

## T-cell metabolism

Metabolism powers T cells by providing the cellular energy and biochemical molecules needed for their proliferation, cytokine production, and cytotoxic activity. During each differentiation stage, T cells adjust their metabolism to meet biosynthetic and energetic demands. T_N_ primarily have slow glycolysis and mainly rely on oxidative phosphorylation (OXPHOS) and fatty acid oxidation, but undergo a metabolic shift from OXPHOS to aerobic glycolysis as they become T_EFF_ [107–110]. After antigen clearance, the memory T cells that switched from T_EFF_, including T_SCM_, T_CM_ and T_EM_, return to relying on OXPHOS, while T_EM_ can rapidly shift to high glycolytic activity, supporting the effector function [107, 108]. In addition to glucose, T cells rely on extracellular amino acids, such as glutamine, serine, and proline, for proliferation, activation, and effector functions [111–113].

The metabolic phenotypes and memory status of T cells are tightly interconnected, and metabolism could shape memory subsets and functions of CAR-T cells [114]. For instance, Fraietta et al. observed that anti-CD19 CAR-T_EFF_ cells from partial-responders and non-responders with CLL exhibited elevated expression of aerobic glycolysis genes and increased uptake of a glucose analog. Furthermore, inhibition of glycolysis using 2-deoxy-D-glucose resulted in increased frequencies of CAR-T_CM_ [23]. Another study documented that T-cell expansion under acidic conditions promoted stem-like T cells [115]. Consistently, recent research has indicated that CAR-T cells maintained at a lower range of normal pH at the beginning of the manufacturing process show enhanced T-cell proliferation and reduced glycolysis [116]. Moreover, IL-7 and IL-15 have been shown to facilitate triglyceride storage, thereby providing T_MEM_ with a stable and long-term energy supply [117]. CAR-T_N_ and CAR-T_CM_ cultured with IL-7/IL-15 demonstrated superior expansion compared with those cultured with IL-2 [86].

Advances in genome editing have also helped overcome metabolic restriction in tumor microenvironments. For instance, Fultang et al. engineered CAR-T cells to resist arginine depletion by knocking in the genes encoding the arginine resynthesis enzymes argininosuccinate synthase and ornithine transcarbamylase [118]. The insertion of both genes increased CAR-T cells’ proliferation and improved their ability to clear leukemia and solid tumors in vivo [118]. Similarly, Ye et al. used a genome-scale gain-of-function CRISPR screen to identify the enzyme proline dehydrogenase 2 (PRODH2], a key enzyme in the proline catabolism pathway [113]. They showed that overexpressing PRODH2 in CAR-T cells promoted memory formation and enhanced cytotoxic activity in leukemia, multiple myeloma, and breast cancer models, both in vitro and in vivo [113].

Additionally, recent evidence suggests that CAR structure may impact the metabolic program in CAR-T cells. A study demonstrated that the CD28 costimulatory domain acts through the PI3K-AKT pathway to increase glucose uptake and glycolysis in response to T-cell activation and differentiation [119]. In contrast, the 4-1BB domain was found to activate both glucose and fatty acid metabolism via the liver kinase B1-AMP-activated protein kinase-acetyl-CoA carboxylase signaling pathway, and it also promotes mitochondrial biogenesis through the PGC1α-mediated pathway [120, 121]. These distinct signaling activities of costimulatory domains, at least in part, drive the T cells carrying CARs with the 4-1BB domain towards T_CM_, whereas the presence of CD28 domains yields T_EFF_ [57]. Importantly, the mere expression of CARs can increase proliferation and metabolic activity [122], and different single-chain variable fragments can lead to various rates of glucose and amino acid consumption in CAR-T cells [122].

## TCR repertoire

TCR is a lineage-defining heterodimeric transmembrane receptor, which is central to initiating ligand-dependent activation of T cells. The diversity of the TCR repertoire is primarily generated by random VDJ recombination of separated gene segments, imprecise joining at gene junction sites, and different pairing of TCRα and TCRβ chains [123]. The estimated lower limit to human TCRαβ repertoire diversity stands at 2.5 × 10^7^ to 1 × 10^8^ [ref 123, 124]. This process results in a varied T-cell population with a highly diverse TCR repertoire poised to respond to foreign antigenic peptides presented by MHC molecules.

TCR repertoire can be used as a surrogate for the T-cell clonotype and expansion [125]. Cancer studies have reported that the diversity and clonality of the TCR repertoire have significant potential in predicting the treatment responses and survivals [126–135]. In studies of peripheral blood TCR repertoire, higher baseline TCR diversity has been reported to correlate with better immunotherapy response [126, 131, 132], and longer progression-free survival (PFS) in both solid and blood malignancies [126, 129, 132, 133]. Additionally, increased TCR clonality after immunotherapy has been associated with improved treatment responses and may serve as predictor for longer PFS [126, 129, 134]. Meanwhile, studies focusing on the TCR repertoire within the tumor microenvironment have shown that the high and evenly distributed TCR diversity at baseline predicts superior survival [127, 130, 135, 136], whereas pre-treatment high TCR clonality is associated with better response to checkpoint blockade therapies [136]. Recently, further studies have indicated that changes of peripheral blood TCR repertoire could serve as a biomarker for disease progression in advanced lung and bladder cancer, and even for the early detection of ovarian cancers [137–139].

In CAR-T therapy, similar power of the TCR repertoire in predicting therapeutic responses and prognosis is observed in CAR-negative T cells [140–142]. A study utilizing TCR sequencing and CITE-seq/transcriptome analysis in MM showed that greater baseline TCR diversity was associated with longer PFS, while increased clonal expansion of terminally differentiated T cell clones correlated with shorter PFS [140]. A follow-on study revealed that MM patients achieving CR harbored higher counts of T cell clonotypes both before and after CAR-T cell infusion compared to those of non-CR patients [141]. However, research exploring TCR diversity in CAR-T cells remains limited. Rade et al. found that hyperexpanded CAR-T cells (>100 cells) were predominantly CD8^+^, and observed the coexistence of CAR-T and non-CAR-T cells with the same clonotype after CAR-T cell infusion [141]. Ledergor et al. revealed a more evenly distributed TCR clonality (lower Gini index) in CAR-T cells compared with non-CAR-T cells [143]. The same trend was found by another study reporting that most CAR-T cells in bone marrow after infusion in MM patients were dominated by a single clone [140]. Despite these findings, the specific clinical implications of TCR repertoire diversity and clonality remain to be fully elucidated.

## Conclusion

Since its first clinical application in 2010, CAR-T cell therapy has shown notable success in the treatment of B-cell malignancies. However, achieving sustainable long-term response and remission remains a challenge, for resistance and disease relapse persist as common outcomes for many patients. T cells are the main adaptive immune cells against tumors and serve as the cellular basis for CAR-T cell therapy. Better exploration and characterization of T-cell properties, such as exhaustion, memory status, subsets, senescence, metabolism, and TCR repertoire will enable the dissection of CAR-T cell function in preclinical models and patients.

Importantly, recent developments in multi-omics profiling techniques, including genomics, transcriptomics, proteomics and beyond, have greatly facilitated the exploration of the remarkable diversity of T-cell phenotypes and expanded our collective knowledge in CAR-T cell therapy. It is to be expected that our growing understanding of T-cell characteristics will allow the optimization of CAR-T cell manufacturing, the prediction of treatment responses, and the personalization of therapy.
